# Editorial: Mobile genetic elements as dissemination drivers of multidrug-resistant Gram-negative bacteria

**DOI:** 10.3389/fcimb.2023.1180510

**Published:** 2023-03-17

**Authors:** Carolina Silva Nodari, Andrés Opazo-Capurro, Santiago Castillo-Ramirez, Vittoria Mattioni Marchetti

**Affiliations:** ^1^ Unité des Bactéries Pathogènes Entériques, Département de Santé Globale, Institut Pasteur, Paris, France; ^2^ Laboratorio de Investigación em Agentes Antibacterianos, Facultad de Ciencias Biológicas, Universidad de Concepción, Concepción, Chile; ^3^ Programa de Genómica Evolutiva, Centro de Ciencias Genómicas, Universidad Nacional Autónoma de México, Cuernavaca, Mexico; ^4^ Department of Microbiology, Faculty of Medicine, University Hospital in Pilsen, Charles University, Pilsen, Czechia; ^5^ Unit of Microbiology and Clinical Microbiology, Department of Clinical-Surgical, Diagnostic and Pediatric Sciences, University of Pavia, Pavia, Italy

**Keywords:** antimicrobial resistance (AMR), high-risk clones, *Enterobacterales*, *Pseudomonas*, *Acinetobacter*, horizontal gene transfer, mobilome

Antimicrobial resistance (AMR) is a global public health threat, which is estimated to cause over a million deaths worldwide every year (Antimicrobial Resistance Collaborators, 2022). The World Health Organization’s critical priority list for the research and development of novel antimicrobial agents is solely composed of multidrug-resistant (MDR) Gram-negative bacilli (GNB), namely *Acinetobacter* spp., *Pseudomonas* spp., and *Enterobacterales* (Tacconelli et al., 2018). Considering the population structure of the aforementioned groups, it is usual to observe the emergence and rapid expansion of some clones, many of which harbour AMR determinants that are encoded by mobile genetic elements (MGEs). Important examples include KPC-producing *Klebsiella pneumoniae*, CTX-M-15-producing *Escherichia coli*, and OXA-23-producing *Acinetobacter baumannii* (Baker et al., 2018). Very often, clinically relevant antibiotic resistance genes are encoded in plasmids and transposons (Partridge et al., 2018). Despite the clinical importance of some MGEs, much is still unknown about their role in the establishment and spreading of high-risk clones.

The main aim of this Research Topic was to shed some light on the potential links between the acquisition of MGEs and the expansion of MDR clones among GNB. We wanted to understand the evolution of MGEs over time, their integration into the genomes of different bacterial species, and their contribution to the selection and spread of some important and highly-resistant clones.

The four original research articles published on this Research Topic confirm the diversity of MGEs associated with AMR and the variety of GNB species where they can reside. García et al. characterized *P. aeruginosa* strains carrying a 54 kbp plasmid with two copies of Tn*4401*, the main transposon associated with the dissemination of the carbapenemase genes *bla*_KPC_ (Naas et al., 2008). Interestingly, in strain PA-2, one of the copies of Tn*4401* harboured *bla*_KPC-33_ instead of the classic *bla*_KPC-2_, a variant associated with resistance to ceftazidime/avibactam (CAZ/AVI) due to the presence of an amino acid substitution (D179Y) in the encoded protein (Compain and Arthur, 2017). That mutation seems to have occurred during CAZ/AVI treatment since PA-1, a CAZ/AVI-susceptible *P. aeruginosa* strain recovered from the same patient before the beginning of treatment, was 99.99% identical to PA-2 but carried two copies of *bla*_KPC-2_-harbouring Tn*4401*. Finally, the authors suggest that the presence of an additional copy of Tn*4401* carrying *bla*_KPC-2_ was not the only mechanism responsible for the maintenance of carbapenem resistance in *P. aeruginosa*, which is usually decreased in KPC-33-producing *Enterobacterales* (Shields et al., 2017). Instead, it could be a combination of various determinants, as they observed a similar phenomenon in another *P. aeruginosa* that, to the best of their knowledge, did not produce KPC-2 (Wozniak et al., 2019).

Kumkar et al. investigated *A. baumannii* isolates from India and used *in silico* tools to characterize resistant determinants, virulence factors, and MGEs. They compared the genome sequences obtained from five isolates recovered in 2005 with 42 complete genomes publicly available. Their analysis confirmed the predominance of International Clone 2 (IC2 – ST2 Pasteur MLST scheme) as a globally distributed high-risk clone (Hamidian and Nigro, 2019) and highlighted an emerging clade belonging to ST622 Pasteur MLST scheme, which was previously described only in Nepal (Shrestha et al., 2015). As expected, the isolates harbored a wide arsenal of intrinsic and acquired AMR determinants, with most isolates recovered between 2014 and 2020 presenting between five and 13 acquired AMR genes. Such genes were associated with a variety of MGEs, including transposons, insertion sequences (ISs), and plasmids. The authors also identified the presence of *A. baumannii* resistance islands (AbaRI) in the chromosomes of 65.6% of the isolates, and the majority of them harbored either AbaR4a or AbaR4b variants (n=14 each). While both AbaRIs carried the carbapenemase encoding gene *bla*_OXA-23_, AbaR4a also harbored resistance determinants to streptomycin and tetracyclines, namely *strAB* and *tetB* genes, expanding the MDR profile of the isolates. The study also indicated the presence of virulence-associated genes that encoded proteins with adherence, iron uptake, and biofilm formation functions.

The study by Tian et al. also explored the virulence potential of an MDR GNB, notably an OXA-232-producing *K. pneumoniae* from China that harbored a virulence plasmid carrying *rmpA2*. Using *in vitro* and *in silico* methodologies, they identified the resistance and virulence determinants of isolate KPTCM and established its genetic relationship with other 45 OXA-232-producing *K. pneumoniae* isolates and with 109 isolates belonging to ST15. Their results revealed that the isolate harbored nine different plasmids, and four of them carried AMR genes, namely *catB*, *arr-2*, *rmtF*, *dfrA14*, *qnrB1*, *strAB*, *bla*_SHV-12_, *sul2*, *bla*_TEM-1B_, and *bla*_OXA-232_. Moreover, this isolate showed a copy of *bla*_CTX-M-15_ in its chromosome, as part of Tn*2012*, and mutations in the quinolone resistance-determining region (QRDR) of *gyrA* (S83F) and *parC* (S80I). The largest of the plasmids present in isolate KPTCM (pKPTCM-1; 167,179bp) carried the virulence gene cluster *iucABCD-iutA* and a copy of *rmpA2* with a frameshift mutation, resulting in a premature stop codon. The absence of a functional *rmpA2* gene could partially explain the  bsence of hypermucoviscosity or hypervirulent phenotype in the *Galleria mellonella* infection model. The phylogenetic comparison of KPTCM with other isolates revealed that it belonged to an ST15 clade that seems to be restricted to China. In fact, this ST was only identified among Chinese isolates when compared to other globally distributed OXA-232-producing *K. pneumoniae*, suggesting an independent acquisition of the *bla*_OXA-232_-harboring plasmid.

Like *bla*_OXA-232_, *bla*_OXA-181_ is another *bla*_OXA-48_-like carbapenemase encoding gene that is frequently present in plasmids. Using an *in silico*-only approach, Yu et al. proposed to compare the 81 *bla*_OXA-181_-positive plasmids available in the NCBI RefSeq database (O’Leary et al., 2016) regarding their host range, conjugative machinery, and the genetic context where *bla*_OXA-181_ was inserted. The authors found that even though almost 90% of the plasmids were found in *E. coli* or *K. pneumoniae* (59.26% and 30.10%, respectively), they could also be observed in other species of the *Enterobacterales* order, such as *Enterobacter hormaechei* and *Morganella morganii*. The plasmids showed large variations in length (from 6 to 123 kbp) and 75/81 carried a ColKP3 replicon. Interestingly, 64 of ColKP3-type plasmids also presented an IncX3-type replicon, which was found to be conjugative by *in silico* analysis. Additionally, 63 of the 66 IncX3-type plasmids carried at least one other AMR gene, namely *qnrS1*, which confers resistance to nalidixic acid and can lead to fluoroquinolone resistance when associated with the presence of mutations in the QRDR region (Ruiz, 2019). In some plasmids, up to six other resistance determinants were identified, conferring resistance to a wide range of antimicrobial classes, including aminoglycosides, sulfamethoxazole/trimethoprim, and chloramphenicol. The authors also describe the genetic context of both *bla*_OXA-181_ and *qnrS1* among the plasmids and highlight the importance of transposons for their mobilization.

The characterization of important antimicrobial-resistant pathogens was not the only feature that linked the studies published as part of this Research Topic. All of them took advantage of genomic tools to properly identify the MGEs associated with AMR phenotypes and to establish phylogenetic relationships between either clinical strains or plasmids. The high throughput sequencing technologies have proven to be a valuable asset for clinical microbiology and AMR surveillance (Boolchandani et al., 2019; Sherry et al., 2023) and will continue to provide essential data for the identification of high-risk bacterial clones and frequently transferable MGEs.

## Author contributions

All authors acted as Editors for this Research Topic. CSN wrote the initial draft, and all authors contributed to and edited the final version of this Editorial.
